# The Influence of Pore Size on the Photocatalytic and SERS Performance of Nanoporous Au–Ag Shells

**DOI:** 10.3390/molecules30071475

**Published:** 2025-03-26

**Authors:** Wenpeng Yang, Wenguang Geng, Xiyuan Lu, Lihua Qian, Shijun Luo, Rui Zheng, Lei Xu, Dapeng Yang

**Affiliations:** 1 College of Electronics and Engineering, North China University of Water Resources and Electric Power, Zhengzhou 450046, China; gengwenguang710@gmail.com (W.G.); luxiyuan@ncwu.edu.cn (X.L.); luoshijun@ncwu.edu.cn (S.L.); zhengrui@ncwu.edu.cn (R.Z.); xulei@ncwu.edu.cn (L.X.); yangdapeng@ncwu.edu.cn (D.Y.); 2 School of Physics, Huazhong University of Science and Technology, Wuhan 430074, China; lhqian@hust.edu.cn

**Keywords:** nanoporous Au–Ag shells, pore size modulation, SERS, LSPR

## Abstract

Nanoporous metals have garnered significant attention in catalysis due to their unique three-dimensional interconnected network structure and pronounced localized surface plasmon resonance (LSPR) properties. In this study, nanoporous Au–Ag shells with varying pore sizes (8, 10, 12, and 18 nm) were synthesized, and their catalytic efficiencies were systematically evaluated. The conversion of p-nitrothiophenol (PNTP) to dimercapto-azobenzene (DMAB) was used to investigate the influence of pore size on the reaction kinetics and surface-enhanced Raman scattering (SERS) effects. Experimental results reveal that the nanoporous Au–Ag shells with a 12 nm pore size exhibit relatively high catalytic efficiency. Furthermore, tuning the pore size enables the modulation of LSPR in the near-infrared region. These findings highlight the critical role of pore size modulation in determining the photocatalytic performance of nanoporous metallic materials and provide valuable insights for the design and optimization of highly efficient photocatalysts.

## 1. Introduction

Single-metal nanoparticles often face inherent trade-offs between their plasmon-enhanced properties and catalytic activities [1]. For instance, small-sized gold nanoparticles exhibit vigorous catalytic activities but generate weak Raman scattering signals, whereas larger gold nanoparticles demonstrate excellent SERS performance but significantly reduced catalytic efficiency [2]. In contrast, nanoporous metallic materials, with their unique three-dimensional interconnected network structures [3], high specific surface areas [4,5], and pronounced LSPR effects [6,7], hold great promise for applications in catalysis [8], SERS [9,10,11,12,13], and sensing technologies [14,15]. Particularly noteworthy are bi-metallic and multi-metallic composite materials, such as Ag–Pt alloy shells on Au nanorods [16], porous Au–Ag composite nanostructures [17], Fe-doped Sn nanoparticles [18], PtNi–Au alloy [19], Ag and Ni co-doped Cu [20], and RuNi alloy [21]. These materials leverage their unique geometric structures and synergistic effects to achieve enhanced catalytic efficiency and superior SERS performance. Furthermore, nanoporous metallic materials can further improve catalytic performance by optimizing the distribution of the local electric field through pore size modulation [22,23,24]. This pore-size tuning strategy provides a valuable approach for designing highly efficient catalytic materials.

In recent years, significant attention has been devoted to optimizing preparation strategies [25,26,27,28,29,30]. While some studies have explored the plasmonic properties and SERS performance of metallic nanomaterials by manipulating parameters such as pore size, geometric structure, and gold-to-silver ratio, these studies still have certain limitations. For example, the survey by Srinoi, P. et al. shows that SiO_2_ shell layers contribute to photocatalytic stability, but their durability has not been fully assessed due to the lack of experimental data on long-term light exposure [31]. Moreira, M. et al. have focused on smaller sized (4–10 nm) nanoparticles, whereas the plasmonic properties and SERS performance of larger nanoparticles (>10 nm) remain insufficiently explored [32]. Similarly, although Kautsar, D.B. et al. successfully synthesized porous three-way catalyst (TWC) particles, their performance has not been systematically compared with other porous structures, leaving their potential advantages unverified [33]. Additionally, the continuous flow synthesis method proposed by Jellicoe, M. et al. and the dealloying method employed by Ortolani, M. et al. have demonstrated feasibility in modulating nanomaterial morphology. However, further optimization of synthesis parameters is required to achieve more precise morphology control and improved performance [34,35]. These limitations highlight the need for more systematic and in-depth research on the optimization and application of metallic nanomaterials. Furthermore, studies investigating the relationship between pore size and catalytic performance in nanoporous Au–Ag shells remain limited, particularly regarding the mechanisms by which pore size modulation affects LSPR and catalytic efficiency.

This study focuses on synthesizing nanoporous Au–Ag shells (NPASs) with tunable pore sizes by adjusting the etching duration, aiming to investigate the impact of pore size on catalytic performance. Utilizing NPASs as an efficient plasmonic catalyst, the primary objective is to examine the dynamic processes involved in reducing p-nitrothiophenol (PNTP) to dimercaptoa-zobenzene (DMAB) on their surface. Furthermore, the study explores the catalytic properties of NPASs with different pore sizes in the context of photocatalysis. By modulating the pore size, the localized surface plasmon resonance (LSPR) in the near-infrared region can be adjusted, which is expected to enhance catalytic efficiency. Ultimately, this research aims to provide deeper insights into the mechanisms of pore size modulation in nanoporous metallic materials and evaluate their potential applications in catalysis.

## 2. Results and Discussions

### 2.1. Microstructure and Composition Analysis of NPASs

**Figure 1:**
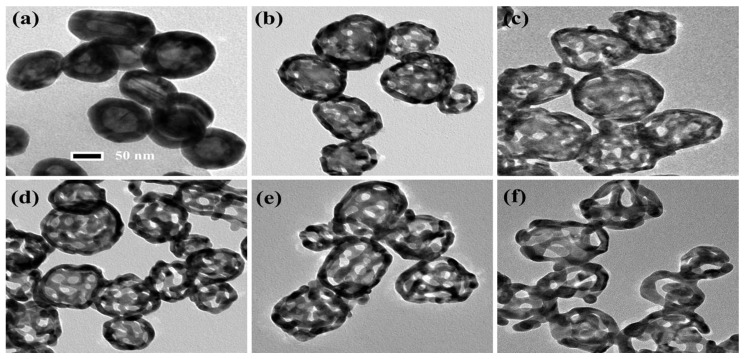
TEM images of ANSs corroded in concentrated nitric acid for 0.5–20 min. (**a**) TEM image of uncorroded ANSs. (**b**–**f**) TEM images corresponding to etching times of 0.5, 1, 5, 10, and 20 min, respectively, with on-shell pore diameters of approximately 6 nm, 8 nm, 10 nm, 12 nm, and 18 nm in that order.

By using a chemical dealloying method on Au–Ag alloy nanoshells (ANSs), NPASs were successfully synthesized. Figure 1 presents the transmission electron microscopy (TEM) images of ANSs subjected to varying etching durations (ranging from 0 to 20 min) in concentrated nitric acid, illustrating the dynamic evolution of the particles from solid to porous structures. As Figure 1a shows, before etching the particles exhibit an intact solid structure with no discernible porosity. Figure 1b–f displays the TEM images corresponding to etching durations of 0.5, 1, 5, 10, and 20 min, respectively. As the dealloying time is extended to 0.5, 1, 5, 10, and 20 min, the pore diameters on the shells are approximately 6, 8, 10, 12, and 18 nm, respectively. For clarity, these samples are designated as NPAS0.5, NPAS1, NPAS5, NPAS10, and NPAS20, based on their etching times. Notably, as shown in Figure 1e, at an etching duration of 10 min NPAS10 exhibits a uniform pore size distribution, and the particles develop a hollow interior structure, resulting in an ideal NPAS. As shown in Figure 1f, when the etching time is extended to 20 min, excessive etching leads to the deterioration of the porous structure in some particles, rendering the porous framework unstable. Therefore, precise control of the etching duration is a critical strategy for optimizing the porous structure.

**Figure 2:**
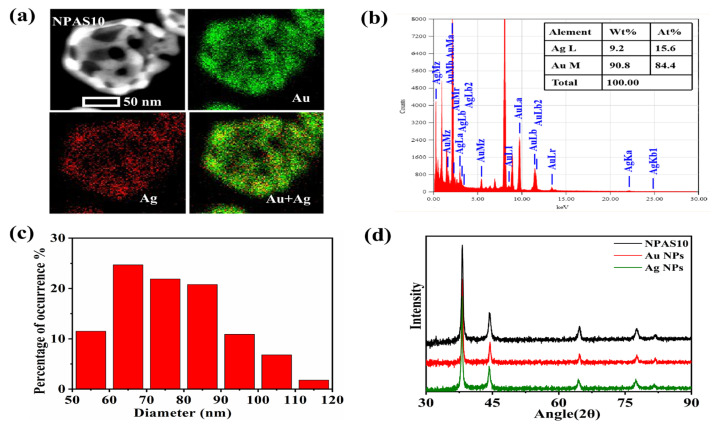
(**a**) EDS elemental mapping of NPAS10, (**b**) EDX analysis, (**c**) particle size distribution, and (**d**) XRD map of NPASs.

As shown in Figure 1e, NPAS10 exhibits a more interconnected pore structure, while the shell thickness is further reduced, forming a uniform and stable porous framework. Therefore, NPAS10 is selected for further detailed characterization to explore its microstructural features and performance advantages. Figure 2a, obtained through energy dispersive X-ray spectroscopy (EDS), illustrates the morphology and elemental distribution of NPAS10, clearly showing the uniform distribution of Ag and Au on the ligaments. As shown in Figure 2b, the elemental concentration ratio of NPAS10 was analyzed using energy-dispersive X-ray spectroscopy (EDX), revealing that the atomic contents of Au and Ag are 84.4 at.% and 15.6 at.%, respectively. Figure 2c depicts the morphological and dimensional characteristics of NPAS10 particles. The particle size distribution was determined by analyzing 1000 particles using Nano Measurer software (version 1.2.5). The results indicate that the NPAS10 particles exhibit an average outer diameter of 80.2 ± 5.4 nm (mean ± standard deviation, SD), a shell thickness of 9.8 ± 1.3 nm, and a hollow cavity diameter of 59.7 ± 4.2 nm. As shown in the X-ray diffraction (XRD) pattern in Figure 2d, NPAS10, along with pure Au and pure Ag nanoparticles, exhibits typical face-centered cubic (fcc) crystal structure characteristics. Distinct diffraction peaks appear at approximately 38°, 44°, 64°, and 77° for each sample, corresponding to the (111), (200), (220), and (311) crystal planes of the fcc lattice structure, respectively. Notably, at the strongest (111) peak (around 38°), the diffraction peak of the NPAS10 sample is positioned between those of Au APs and Ag NPs. It is slightly shifted to a higher angle (approximately 38.3°) than pure Au nanoparticles (approximately 38.2°). This shift to a higher angle indicates a reduction in the lattice parameter of Au due to the incorporation of smaller Ag atoms, leading to a slight lattice contraction. Consequently, it can be confirmed that the nanoporous Au–Ag shells form a homogeneous solid-solution alloying phase rather than a mixture of separate Au and Ag phases. Furthermore, no distinct double peaks or splitting features are observed in the plots, further confirming that the samples exhibit a homogeneous Au–Ag alloy phase structure.

**Figure 3:**
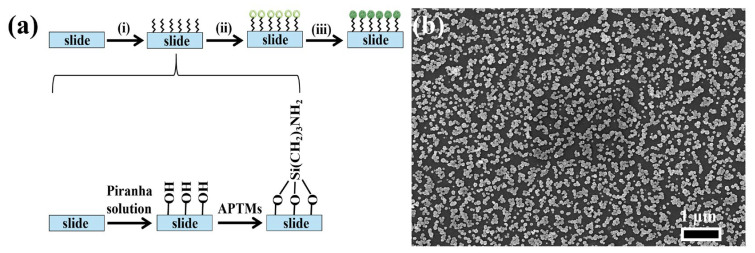
(**a**) SERS substrate preparation process: (i) hydroxylation and silanization, (ii) modified nanomaterials: the surface of the slide was chemically modified with silane molecules to create a substrate with amino functional groups, providing active sites for the attachment of nanoparticles, (iii) Immobilized nanomaterials: Nanoparticles are immobilized on functionalized surfaces through chemical bonding to form substrates that exhibit SERS-enhancing effects; (**b**) SEM image of the NPAS substrate.

Figure 3a illustrates the process of preparing NPAS substrates with different pore sizes, consisting of three key steps. First, the glass slides are thoroughly cleaned to remove organic contaminants and surface residues. This is achieved by washing the slides with detergent, rinsing them with ultra-pure water, and then drying them under a stream of nitrogen gas to ensure a clean surface. The second step involves hydroxylation and silylation of the slides. During hydroxylation, the slides are treated with Piranha solution, a highly reactive mixture of concentrated sulfuric acid (H_2_SO_4_) and hydrogen peroxide (H_2_O_2_). This solution effectively removes organic residues and introduces hydroxyl (-OH) groups onto the glass surface, activating it for further modification. After rinsing with ultra-pure water and drying, the slides are ready for the next functionalization step. Following hydroxylation, the slides undergo silylation by being immersed in a 0.5 M solution of 3-aminopropyltrimethoxysilane (APTMS) for 24 h. During this step, the amino group (-NH_2_) of APTMS reacts with the hydroxyl groups on the slide surface via a condensation reaction, forming stable Si-O-Si bonds. This reaction introduces amine (-NH_2_) functional groups to the surface, significantly enhancing its ability to bind with nanostructures. Finally, ANSs are immobilized onto the functionalized slides. The amine groups on the surface facilitate the attachment of ANSs through electrostatic interactions or covalent bonding, ensuring a uniform and stable deposition of the nanoporous structures on the substrate. Figure 3b shows that NPAS10 particles are uniformly distributed at the micrometer scale with consistent particle sizes. This uniform distribution and porous structure offer a large specific surface area and enhance structural stability, making them well-suited for catalytic applications.

### 2.2. UV–Vis Spectroscopy Analysis and LSPR Properties

**Figure 4:**
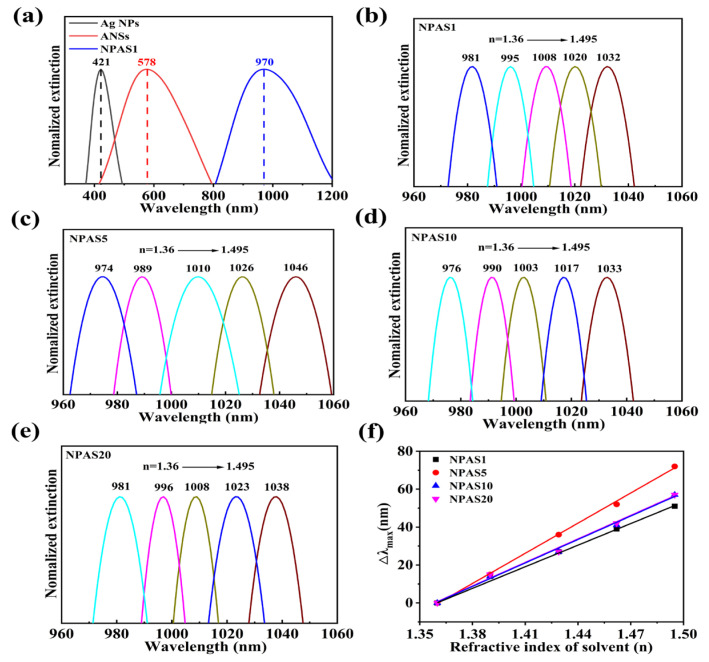
(**a**) UV–Vis spectra of NPAS1, ANSs, and Ag NPs; (**b**–**e**) absorption spectra of NPAS substrates with different pore sizes in solvents with different refractive indices, ethanol: *n* = 1.36, 3:1 mixture of ethanol and toluene (*v*/*v*, *n* = 1.39), 1:1 mixture of ethanol and toluene (*v*/*v*, *n* = 1.429), 1:3 mixture of ethanol and toluene (*v*/*v*, *n* = 1.462), toluene (*n* = 1.495); (**f**) the peak shifts (the Δ*λ*max is relative to the peaks which are obtained in the ethanol dispersant) of NPASs with different pore sizes vary with the refractive index (*n*) of the surrounding medium. The sensitivity coefficients of NPAS1, NPAS5, NPAS10, and NPAS20 are 370.8, 529.3, 411.5, and 411.3 nm/RIU, respectively. All spectra were normalized by their peaks.

Based on the above structural and morphological analyses, further evaluation of the potential of NPASs with different pore sizes for optical and catalytic applications is conducted. The UV–Vis absorption spectra of Ag NPs, ANSs, and NPAS1 substrates were measured in ultra-pure water using a UV–Vis spectrophotometer to determine their LSPR peaks [36,37,38]. The measurement range for Ag NPs and ANSs is 300–900 nm, while NPASs in different solvents are 400–1100 nm. To better illustrate the variation in the peak positions of the nanomaterials, only the narrower regions are displayed in the figure. Normalization of the Ag NPs, ANSs, and NPAS1 peaks yields in the UV–Vis spectrum is shown in Figure 4a. Their LSPR peaks are located at 421 nm, 578 nm, and 970 nm, respectively. As shown in the figure, the peak of Ag NPs at 421 nm disappears after the reaction between Ag NPs and AuCl_4_^−^, indicating that the Ag NPs have been completely consumed. After the ANSs undergo corrosion, their LSPR peaks vanish, and a new peak emerges at 970 nm. As the corrosion time increases, the position of the new peak changes slightly. For instance, the LSPR peaks of NPAS5, NPAS10, and NPAS20 are located at 958 nm, 963 nm, and 968 nm, respectively. These results suggest that, compared to traditional metals such as gold and silver, porous structures absorb light more effectively in the near-infrared region [39].

Absorbance tests were conducted by sequentially immersing the NPAS substrates in solvents with varying refractive indices to obtain UV–Vis spectra [40]. As shown in Figure 4b, the LSPR peak of NPAS1 undergoes a significant red-shift as the solvent’s refractive index gradually increases from 1.36 to 1.495. Similarly, in Figure 4c–e, the LSPR peak positions of NPAS5, NPAS10, and NPAS20 vary with the refractive index in the same manner as NPAS1. Furthermore, Figure 4f demonstrates that the relative peak displacement (Δ*λ*max) with respect to the sample’s position in ethanol exhibits a linear relationship with the refractive index of the solvent for all samples. The slope is determined through linear fitting to calculate the sensitivity coefficients, which are 370.8, 529.3, 411.5, and 411.3 nm/RIU for NPAS1, NPAS5, NPAS10, and NPAS20, respectively.

The experimental results also reveal that ANSs are insensitive to the refractive index of the solvents, as their LSPR peak positions remain unchanged when the refractive index varies. In contrast, the sensitivity of NPASs to the refractive index of solvents may be attributed to the dielectric properties of their porous structures. Additionally, the sensitivity coefficient of NPASs increases with increasing pore size, reaching a maximum when the pore size is 10 nm. Beyond this point, it decreases as the pore size continues to grow, eventually stabilizing. NPASs with high sensitivity can be utilized for developing LSPR nano-sensors.

### 2.3. SERS Enhancement Effect and Catalytic Activity Analysis

The SERS technique has been successfully utilized as a highly sensitive molecular detection tool for monitoring chemical and physical changes in plasma surfaces over time [9,10,11,12,13]. Furthermore, NPASs have been demonstrated to exhibit excellent chemical catalytic activity and SERS performance [41]. To investigate the effect of pore size on photocatalytic kinetics, NPAS substrates with different pore sizes were immersed in a 0.1 mM PNTP ethanol solution for two hours. The substrates were then removed and rinsed with ultra-pure water to eliminate physically adsorbed PNTP molecules. Finally, a layer of chemically adsorbed PNTP molecules was self-assembled on the surface of NPAS substrates to evaluate their SERS and catalytic performance.

**Figure 5:**
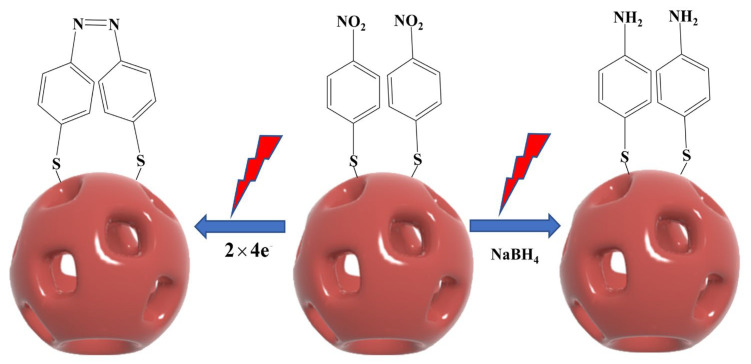
Reaction mechanism of PNTP molecules coupled to generate DMAB molecules under plasma-driven photocatalytic conditions and schematic diagram of the catalytic generation of PATP in the presence of NaBH_4_.

The conversion of PNTP to DMAB can occur through photocatalytic and chemical reduction. Figure 5 presents a schematic diagram illustrating the photocatalytic reaction of PNTP on the surface of NPAS substrates and the sodium borohydride (NaBH_4_) reduction reaction for its conversion to DMAB.

In the chemical reduction process, NaBH_4_ is typically used as an external reducing agent. First, PNTP is reduced to p-aminothiophenol (PATP) in the presence of NaBH_4_. Then, under oxidizing conditions, two PATP molecules undergo a coupling reaction, ultimately producing DMAB. In contrast, the photocatalytic reaction does not require an external chemical reductant. Instead, PNTP conversion is directly driven by the thermoelectrics generated through LSPR. In this process, PNTP molecules first adsorb onto the surface of NPAS substrates via Au–S or Ag–S bonds. Under laser irradiation, NPAS substrates generate LSPR, inducing electron mobility and producing high-energy hot electrons and hot holes. These hot electrons are then transferred to the nitro group (-NO_2_) of PNTP, triggering electron transfer and molecular rearrangement. This process ultimately facilitates the reduction of PNTP and the formation of an azo bond (-N=N-), leading to the direct generation of DMAB. As the reaction progresses, the DMAB product desorbs from the catalyst surface, completing the catalytic cycle.

In this process, two PNTP molecules accept eight electrons and eight protons, undergoing reductive transformation to form DMAB while generating four water molecules. This photocatalytic process can be expressed as:

2PNTP + 8e^−^ + 8H^+^ → DMAB + 4H_2_O

where 8H^+^ represents protons (H^+^) which help maintain charge balance during the reduction process and facilitate the electron transfer necessary for the conversion of PNTP into DMAB. Unlike semiconductor photocatalysis, where photogenerated holes participate in oxidation reactions, in this plasmon-driven process, hot holes generated by LSPR are rapidly neutralized by electron backfilling within the metallic nanostructure. Consequently, oxidative side reactions are minimized.

Notably, in the plasma-driven photocatalytic reaction, the nitro group (-NO_2_) of PNTP can be efficiently reduced without additional chemical reductants, relying solely on the hot electrons generated by LSPR to achieve selective catalytic conversion. Among them, hot electrons are directly involved in the reduction of PNTP, while hot holes catalyze the oxidation of water. Specifically, water molecules are oxidized by hot holes to produce protons (H^+^) and oxygen (O_2_), making oxygen the primary oxidation product [42].

**Figure 6:**
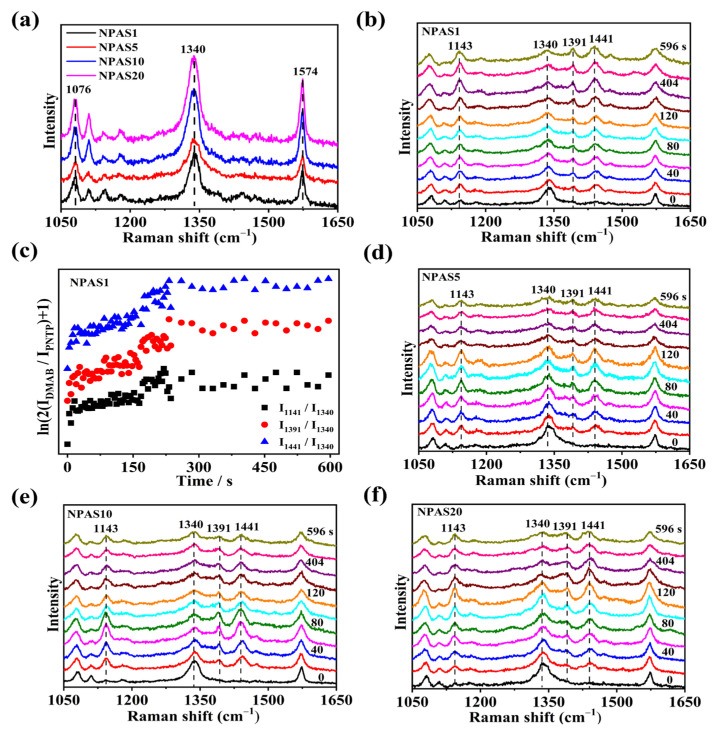
(**a**) SERS spectra of PNTP molecules on NPAS substrates with different pore sizes; (**b**) SERS spectra of PNTP molecules on NPAS1 substrate under continuous 633 nm laser irradiation; (**c**) Variation of ln(2*I_DMAB_/I_PNTP_* + 1) with time at different peaks during plasma-driven PNTP coupling to DMAB on NPAS1 substrate. The SERS spectra of PNTP molecules on (**d**) NPAS5, (**e**) NPAS10, and (**f**) NPAS20 are collected at different times under continuous 633 nm laser irradiation. The integration time is 4 s, and the laser power on the sample is 0.17 mW.

The SERS spectra of PNTP molecules on NPAS substrates with different pore sizes were collected using a laser with a wavelength of 633 nm. The laser power on the sample was set to 0.17 mW, with a collection time of 4 s. As shown in Figure 6a, the SERS spectrum of PNTP molecules exhibits three prominent peaks at 1076 cm^−1^, 1340 cm^−1^, and 1574 cm^−1^, corresponding to C-S stretching, O-N-O stretching, and benzene ring vibration modes, respectively. The Raman enhancement of PNTP molecules on NPAS10 and NPAS20 substrates is slightly greater than that on the NPAS1 and NPAS5 substrates. Figure 6b presents the SERS spectra of PNTP molecules on NPAS substrates as a function of illumination time. After the laser irradiates samples, new Raman peaks appear at 1141 cm^−1^, 1391 cm^−1^, and 1441 cm^−1^, corresponding to C-N symmetric stretching, N=N stretching, and C-H in-plane bending vibration modes, respectively. These new peaks are characteristic peaks of DMAB molecules. As the illumination time increases, the Raman peak intensity of DMAB molecules gradually increases, meanwhile the intensity of PNTP Raman peaks gradually decreases. This trend indicates that PNTP molecules are being converted into DMAB molecules with continued illumination.

To quantitatively evaluate the catalytic performance of NPAS1, the reaction kinetics of PNTP molecule coupling to form DMAB molecules is analyzed. In Figure 6c, the variation of ln(2*I_DMAB_/I_PNTP_* + 1) over time at 1141 cm^−1^, 1391 cm^−1^, and 1441 cm^−1^ on the NPAS1 substrate is observed. These peaks are typical of the characteristic peaks of the DMAB molecule. As shown in Figure 6c, at first, an increasing number of PNTP molecules are converted into DMAB molecules. After a certain period of illumination, saturation is reached, indicating that the conversion of PNTP to DMAB is nearly complete. The reaction rate constants at the initial stage for various peak positions on the NPAS substrates were determined by analyzing different Raman peaks. For instance, the rate constants at the peaks of 1141 cm^−1^, 1391 cm^−1^, and 1441 cm^−1^ are 0.0016 s^−1^, 0.0023 s^−1^, and 0.0022 s^−1^, respectively. The rate constants of the three characteristic DMAB peaks differ. The rate constant at 1141 cm^−1^ is lower than those at 1391 cm^−1^ and 1441 cm^−1^. One possible explanation is the difference in the breaking and formation of chemical bonds. In other words, different peaks have different processes for the transport and absorption of hot electrons. The SERS spectra of PNTP molecules on NPAS5, NPAS10, and NPAS20 substrates as a function of illumination time are shown in Figure 6d–f. The PNTP molecules undergo a similar transformation on these substrates, gradually coupling into DMAB molecules under laser irradiation.

**Figure 7:**
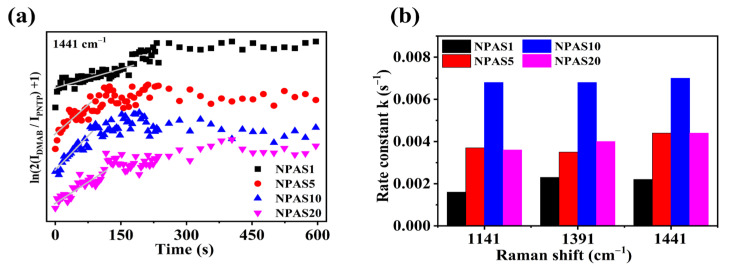
(**a**) The variation of ln(2*I_DMAB_/I_PNTP_* + 1) over time for the Raman characteristic peak of DMAB at 1441 cm^−1^ on NPAS substrates with different pore sizes, and (**b**) the rate constants at different peak positions.

Figure 7a illustrates the variation of ln(2*I_DMAB_/I_PNTP_* + 1) over time at the 1441 cm^−1^ peak. As shown in the figure, on NPAS substrates with different pore sizes ln(2*I_DMAB_/I_PNTP_* + 1) increases with illumination time, indicating that PNTP molecules are gradually converted into DMAB molecules. With prolonged illumination, ln(2*I_DMAB_/I_PNTP_* + 1) stabilizes on NPAS1 and NPAS20 substrates, suggesting that the coupling reaction eventually reaches saturation. However, on the NPAS5 and NPAS20 substrates, after a certain period of illumination ln(2*I_DMAB_/I_PNTP_* + 1) begins to decline, indicating a reduction in DMAB molecules relative to PNTP molecules. This phenomenon is attributed to the photothermal effect, which leads to the desorption of both PNTP and DMAB molecules.

These results suggest that the conversion of PNTP molecules into DMAB molecules is a substrate- and time-dependent plasma-driven surface photocatalytic reaction. Figure 7b presents the reaction rate constants at the initial stage on NPAS substrates with different pore sizes, calculated from various Raman peaks. For NPAS1, the rate constants at the 1141 cm^−1^, 1391 cm^−1^, and 1441 cm^−1^ peaks are 0.0016, 0.0023, and 0.0022 s^−1^, respectively. For NPAS5, they are 0.0037, 0.0035, and 0.0044 s^−1^, respectively. For NPAS10, the rate constants are 0.0068, 0.0068, and 0.0070 s^−1^, While for NPAS20, they are 0.0036, 0.0040, and 0.0044 s^−1^, respectively. These findings indicate that the catalytic rate of NPASs initially increases with the pore size, then decreases as the pore size continues to increase, reaching a maximum at 12 nm. This trend is attributed to the combined influence of optical absorption and the density of catalytically active sites in the porous structure on the kinetic process, as well as the synergistic interaction between the metal components, which enhances the plasmonic activity of Ag and the catalytic activity of Au.

### 2.4. The Influence of Photothermal Effects on Catalytic Kinetics

**Figure 8:**
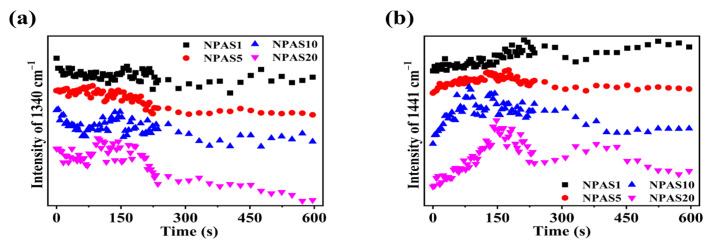
During the photocatalytic reaction of PNTP molecules on NPAS substrates with different pore sizes, (**a**) the relationship between the peak intensity of the O-N-O stretching vibration at 1340 cm^−1^ for PNTP molecules and the illumination time, and (**b**) the peak intensity of the N=N stretching vibration at 1441 cm^−1^ for DMAB molecules with illumination time.

Previously, it was discussed that under prolonged laser irradiation, the reaction of PNTP molecules on the surfaces of NPAS1 and NPAS20 eventually reached saturation. In contrast, a decreasing trend in DMAB yield was observed on NPAS5, with an even more pronounced reduction on NPAS10. From Figure 7b and Figure 6d–f, it can be observed that the SERS peak intensity on NPAS substrates with different pore sizes gradually decreases under prolonged laser excitation. Figure 8a illustrates the variation in the intensity of the characteristic PNTP peak at 1340 cm^−1^ due to illumination time, while Figure 8b presents the variation in the intensity of the characteristic DMAB peak at 1441 cm^−1^ due to illumination time. As shown in the figure, with increasing laser illumination time the peak intensities of both PNTP and DMAB molecules gradually weaken. This indicates that on NPAS substrates with different pore sizes, the amount of PNTP and DMAB molecules decreases with prolonged illumination time, and this reduction becomes more pronounced as the pore size increases. This is due to the heat generated during the photocatalytic reaction, which breaks the Ag–S/Au–S bonds, leading to the desorption of DMAB and PNTP molecules from the surface of the NPASs.

## 3. Materials and Methods

### 3.1. Materials

Hydrogen tetrachloroaurate hydrate (HAuCl_4_·4H_2_O, 99%), silver nitrate (AgNO_3_, 99.8%), p-nitrothiophenol (C_6_H_5_NO_2_S, PNTP, 96%), and ethanol (AR) were obtained from Sinopharm Chemical Reagent Co., Ltd. (Shanghai, China). Trisodium citrate dihydrate (C_6_H_5_Na_3_O_7_·2H_2_O, 99%) was purchased from Sigma (Louis, MO, USA). Nitric acid (HNO_3_, 69%), 3-aminopropyltrimethoxysilane (C_6_H_17_NO_3_Si, APTMS, 99%), hydrogen peroxide solution (H_2_O_2_, 30%), and sulfuric acid (H_2_SO_4_, 98%) were purchased from Aldrich (St. Louis, MO, USA). All chemical reagents were used as received without further purification. Aqueous solutions were prepared using high-purity deionized (DI) water (≥18 MΩ) from an ELGA water purification system.

### 3.2. Synthesis of Ag Nanoparticles (NPs)

All glassware was immersed in aqua regia (a mixture of hydrochloric acid and nitric acid in a 3:1 volume ratio) for one hour, then washed three times with deionized water and dried before use. Ag NPs with a size of approximately 60 nm were synthesized via a hydrothermal approach, utilizing sodium citrate as the reducing agent and AgNO_3_ as the silver source. In a 250 mL three-necked round-bottom flask, 100 mL of a 1 mM AgNO_3_ solution was added and heated to its boiling point (~100 °C) using an oil-bath magnetic stirrer while continuously stirring at 600 rpm. Once the solution reached 100 °C, 1 mL of a 0.034 M trisodium citrate solution was rapidly injected into the boiling AgNO_3_ solution using a pipette. The reaction mixture was maintained at 100 °C under continuous stirring (600 rpm) for 60 min to ensure the complete reduction of Ag^+^ ions. During the reaction, the solution gradually changed to a yellow-green, indicating the formation of Ag NPs. After synthesis, the Ag NPs were collected via centrifugation, thoroughly washed with ultra-pure water to remove residual reactants, and subsequently dried under a nitrogen atmosphere. The resulting silver nanoparticles had an average diameter of approximately 60 nm.

### 3.3. Synthesis of Au–Ag Alloy Nanoshells (ANSs)

ANSs were synthesized via a galvanic replacement reaction utilizing as-prepared Ag NPs as a sacrificial template. Briefly, the as-synthesized Ag colloidal solution was heated to boiling with constant stirring, followed by the simultaneous addition of 0.9 mL of a 50 mM HAuCl_4_ aqueous solution and 0.9 mL of a 100 mM AgNO_3_ aqueous solution with a concentration of 100 mM. Immediately afterwards, 2.1 mL of a 0.034 M sodium citrate solution was added to the mixture, with heating and stirring being sustained throughout the process. The solution quickly became purple before gradually transitioning to reddish brown and then green. The resulting mixture was left at the boiling temperature with vigorous stirring for one hour. Next, the solution was cooled naturally to room temperature with continuous stirring. Then, ANSs with hollow interior, an inner diameter of about 60 nm, and an outer diameter of about 80 nm were obtained. After synthesis, deionized water was added to the solution to adjust the total volume to 105 mL. This dilution process slows the reaction rate by decreasing the concentration of metal ions, thereby preventing overreaction and particle aggregation while ensuring the homogeneity and stability of the nanoparticles.

### 3.4. Preparation of SERS-Active NPAS Substrates

To fabricate SERS-active NPAS substrates, glass slides were initially soaked in Piranha solution (a mixture of concentrated sulfuric acid and hydrogen peroxide in a 2:1 volume ratio) for 30 min. Subsequently, they were placed in an ethanol solution containing 5% APTMS for 24 h. The final step involved immersing the slides in NPASs colloids for 12 h. NPASs were synthesized through chemical dealloying of ANSs. ANSs were subjected to a dealloying reaction in a 69% (*v*/*v*) concentrated HNO_3_ solution. During this process, the alloys underwent selective corrosion at 0 °C, where silver, being more reactive than gold, was preferentially dissolved by the concentrated nitric acid. As silver dissolves, the gold portion remains relatively stable, reaggregates, and forms NPASs with a hollow interior and a porous shell [34,43,44,45]. Each NPAS features a hierarchically porous architecture consisting of a central hollow cavity with a diameter of tens of nanometers, and a shell adorned with ultrafine pores whose size can be tuned by adjusting the dealloying duration, as Figure 1 illustrates. To control the pore size of NPASs, the dealloying time was extended to 1, 5, 10, and 20 min, aiming to obtain NPASs with pore diameters of approximately 8, 10, 12, and 18 nm. For convenience, these samples were designated as NPAS1, NPAS5, NPAS10, and NPAS20, corresponding to their respective etching times. After thorough rinsing with distilled water, the slides were dried under a stream of nitrogen gas. To functionalize the catalyst surface with PNTP, the catalyst-coated substrates were incubated in a 0.1 mM ethanol solution of PNTP for 2 h, then washed with ultra-pure water to remove physisorbed molecules.

### 3.5. Microstructural Characterization and Raman Spectrum

Ultraviolet–visible (UV–Vis) absorption spectra were recorded using a UV-2550 spectrophotometer (Shimadzu, Kyoto, Japan) without any coating applied. The microstructures of the as-prepared materials were characterized using a field-emission SEM (JEM7600F) and a transmission electron microscope (JEOL2100). Both devices were manufactured by JEOL Ltd. In Akishima, Japan. Raman spectra were obtained using a Renishaw inVia Raman microscope and spectrometer. The excitation source was a He–Ne laser (Melles Griot, Carlsbad, CA, USA) with a wavelength of 632.8 nm. The typical acquisition time in this study was 4 s.

## 4. Conclusions

This study systematically analyzed the performance of NPASs in photocatalysis and SERS by tuning their pore sizes. The experimental results demonstrated that NPASs with different pore sizes (8, 10, 12, and 18 nm) exhibit varying catalytic properties. Among them, the relatively high catalytic efficiency of NPAS10 may be attributed to the synergistic effect of surface area, active sites, and the LSPR effect. However, the results also indicate that the catalytic efficiency is not only affected by pore size and that the relationship between the two is not linear. These findings highlight the potential of pore size modulation in optimizing the catalytic performance of NPASs, but further in-depth studies are needed to fully understand the underlying mechanisms.

## Data Availability

The data presented in this study are available upon request from the corresponding author.
